# Burkholderia cenocepacia Strain CEIB S5-1, a Rhizosphere-Inhabiting Bacterium with Potential in Bioremediation

**DOI:** 10.1128/genomeA.00056-15

**Published:** 2015-03-05

**Authors:** Fernando Martínez-Ocampo, Luis Fernando Lozano-Aguirre Beltrán, Armando Hernández-Mendoza, Luis Enrique Rojas-Espinoza, Elida Carolina Popoca-Ursino, María Laura Ortiz-Hernández, Enrique Sánchez-Salinas, Fernando Ramos Quintana, Edgar Dantán-González

**Affiliations:** a Centro de Investigación en Biotecnología, Laboratorio de Investigaciones Ambientales, Universidad Autónoma del Estado de Morelos, Cuernavaca, Morelos, Mexico; b Centro de Ciencias Genómicas, Programa de Genómica Evolutiva, Universidad Nacional Autónoma de México, Cuernavaca, Morelos, Mexico; c Centro de Investigación en Dinámica Celular, Universidad Autónoma del Estado de Morelos, Cuernavaca, Morelos, Mexico; d Facultad de Ciencias Biológicas, Universidad Autónoma del Estado de Morelos, Cuernavaca, Morelos, Mexico

## Abstract

Burkholderia cenocepacia is considered an opportunistic pathogen from humans and may cause disease in plants. A bioprospection from a plaguicide-contaminated agricultural field in Mexico identified several methyl parathion-degrading bacteria. Here, we report the draft genome sequence of B. cenocepacia strain CEIB S5-1, which gave us clues into ecological biodiversity.

## GENOME ANNOUNCEMENT

Burkholderia cenocepacia is an important member of the B. cepacia complex (BCC), a group of human opportunistic pathogens in patients with cystic fibrosis (1), and it is also widely distributed in the environment, especially in the rhizosphere of crop plants (2). In 2012, Popoca-Ursino (3) reported three new bacterial species of the genus Burkholderia isolated from agricultural soil in the state of Morelos, Mexico, and these species have the capability to hydrolyze methyl parathion (MP) and completely degrade *p*-nitrophenol (PNP). From this group, we identified by 16S RNA phylogenetic analysis Burkholderia zhejiangensis strain CEIB S4-3 (4), which completely degraded PNP in 15 h, while B. cenocepacia strains CEIB S5-1 and Burkholderia sp. S5-2 do the same but in 21 h. These results are relevant, because there are few reports of microorganisms capable of simultaneously hydrolyzing MP and degrading PNP (5–7).

Genomic DNA was obtained using the AxyPrep bacterial genomic DNA miniprep kit (Axygen). The genomic DNA was sequenced in the Genome Analyzer IIX system (Illumina). We obtained a random data set of 26,125,416 paired-end reads, each 72 bases in length. Quality-based trimming was performed with a DynamicTrim (SolexaQA++) perl script, and genome assembly was accomplished using the SPAdes (version 3.1.1) program. The draft genome has 177 contigs, with a calculated 8,055,498 bp of total length and an *N*_50_ contig size of 170,699 bp. We aligned the draft genome with 41 complete genomes (chromosomes and plasmids) of the Burkholderia genus using the NUCmer program. The draft genome of B. cenocepacia strain CEIB S5-1 has 92.19, 92.17, 92.12, and 91.82% identity and 6.42, 6.29, 6.69, and 6.15 Mb alignment coverage with the genomes of B. cenocepacia HI2424 (3 chromosomes, 1 plasmid, and 7,702,840 bp in length), B. cenocepacia AU 1054 (3 chromosomes and 7,279,116 bp in length), B. cenocepacia MC0-3 (3 chromosomes and 7,971,389 bp in length), and B. cenocepacia J2315 (3 chromosomes, 1 plasmid, and 8,055,782 bp in length), respectively.

The contigs were analyzed on the RAST server, and 7,390 coding sequences (CDSs) were identified. We also identified the 16S rRNA gene of B. cenocepacia strain CEIB S5-1 using the RNAmmer 1.2 server and a comparison with 101 16S rRNA genes of the Burkholderia genus and two 16S rRNA genes of the Ralstonia genus (outgroup) was made. The sequences were aligned using the MUSCLE server, and a phylogenetic analysis was performed in the MEGA (version 6.1) program with the neighbor-joining algorithm using 1,000 replicates for bootstrapping. Phylogenetic analysis confirms that B. cenocepacia strain CEIB S5-1 is closely related to the B. cenocepacia species.

The 7,390 predicted CDSs were translated and used for a functional analysis with the Clusters of Orthologous Groups (COG) database. The COG database identified 4,327 proteins (58.55%), 1,507 open reading frames (ORFs) (20.39%) with an identified function and 2,820 ORFs (38.16%) with an uncharacterized function. We are investigating the potential genes involved in PNP and PM degradation.

### Nucleotide sequence accession numbers.

This whole-genome shotgun project has been deposited at DDBJ/EMBL/GenBank under the accession no. JTLT00000000. The version described in this paper is version JTLT01000000.
